# Neutrophil Extracellular Traps Reprogram IL-4/GM-CSF-Induced Monocyte Differentiation to Anti-inflammatory Macrophages

**DOI:** 10.3389/fimmu.2017.00523

**Published:** 2017-05-17

**Authors:** Anderson B. Guimarães-Costa, Natalia C. Rochael, Fabiano Oliveira, Juliana Echevarria-Lima, Elvira M. Saraiva

**Affiliations:** ^1^Departamento de Imunologia, Instituto de Microbiologia Paulo de Góes, Universidade Federal do Rio de Janeiro, Rio de Janeiro, Brazil; ^2^Laboratory of Malaria and Vector Research, Vector Molecular Biology Unit, National Institute of Allergy and Infectious Diseases, National Institutes of Health, Rockville, MD, USA

**Keywords:** neutrophil extracellular traps, monocytes, IL-4 receptor, elastase, dendritic cells, *Leishmania*

## Abstract

Monocyte-derived dendritic cells (mo-DCs) are essential for the development of a Th1 protective immune response against *Leishmania* parasites. It is well known that IL-4 and GM-CSF drive differentiation of human monocytes to dendritic cells (DCs). Here, we investigate if neutrophil extracellular traps (NETs) disrupt this process. NETs-enriched supernatants, generated after human neutrophil activation by *Leishmania* promastigotes, were added to monocytes and differentiation monitored by expression of molecules associated with macrophage and DCs phenotypes, cytokine production, and parasite killing. We found that NETs addition to IL-4/GM-CSF-treated monocytes prevented then to fully differentiate into DCs. No effect was observed if NETs were treated with DNase or by filtering the traps. Moreover, NETs closely interact with monocytes and downregulate the expression of the IL-4 receptor, which in turn disrupts fully differentiation of monocytes into DCs. Neutrophil elastase inhibition rescues the monocytes to DCs differentiation. Monocytes cultured with IL-4/GM-CSF and NETs differentiated into macrophages, as observed by the increased expression of CD68, CD32, and CD163, and decreased expression of CD80. Moreover, NET addition to IL-4/GM-CSF-treated monocytes rendered these cells less efficient to kill *Leishmania* parasites. Altogether, our results show that NETs interfere with IL-4/GM-CSF driven differentiation, reprogramming the generation of mo-DCs to an anti-inflammatory macrophage.

## Introduction

Neutrophils are endowed with several antimicrobial proteins and upon activation can kill microorganisms by the release of the neutrophil extracellular traps (NETs) (1). NETs are web-structures composed by DNA and proteins from different neutrophil compartments, such as histones and elastase, functioning in the contention and killing of microorganisms (1, 2). Although the hallmark of NETs is the ability to confine and kill pathogens, many studies have reported that NETs can orchestrate the immune response during autoimmune diseases (3–9). NETs-activated dendritic cells (DCs) induce anti-neutrophil cytoplasmic antibody production and autoimmunity in naïve mice, a feature prevented by NET digestion with DNase (3). NETs were found to be the major inducer of type I interferon (IFN) production by plasmacytoid DCs in patients with systemic lupus erythematosus, meaning that NETs can modulate DCs function (5–7). To date NETs were described to activate both pro-inflammatory and anti-inflammatory responses in other immune cell types. NETs formed upon activation of neutrophils with *Mycobacterium tuberculosis*, interact with and activate macrophages, inducing IL-6, tumor necrosis factor-α (TNF-α), IL-1β, and interleukin-10 (IL-10) production (10), and NETs from cholesterol crystal-stimulated neutrophils prime monocytes to produce IL-6 and IL-1β in response to these cholesterol crystal (11). Contrariwise, NETs inhibit the activation of monocyte-derived dendritic cells (mo-DCs) by lipopolysaccharide (LPS) and promote polarization to a Th2 immune response (12).

During *Leishmania* infection, neutrophils are one of the first lines of defense and the first cells to be parasitized by *Leishmania* parasites (13, 14). Leishmaniasis is a neglected tropical disease transmitted by bites of phlebotomine sand flies, which inoculates parasites along with saliva, rousing a massive and rapid influx of neutrophils, followed by monocytes to the inoculation site, where these cells are likely to interact (13, 14). Our group has demonstrated that *Leishmania* activate NETs formation, and parasites are ensnared and killed by NETs-associated histones (15). However, a portion of the *Leishmania* population, despite its ability to induce NETs release, evade NETs-mediated killing (16–18). Moreover, saliva of the *Leishmania* vector, *Lutzomyia longipalpis*, contains a potent nuclease that digests NETs structure allowing the parasites to escape NET-mediated killing (18). Recently, analysis of 35 biopsies of patients with cutaneous leishmaniasis showed that NETs were presented in 77.1% of the biopsies. Furthermore, amastigotes were observed ensnared by the traps (19).

Depending on the microenvironment, monocytes can differentiate into DCs or macrophages. Dermal infection by *Leishmania major* leads to the differentiation of mo-DCs in the inflammatory site, which are infected by the parasite (20). Interestingly, dermal-differentiated DCs produce large amounts of IL-12 and stimulate a specific T cell response. Moreover, DCs differentiated from monocytes at the site of *Leishmania* infection produce large amounts of nitric oxide (NO), an important mediator for parasite killing (21). Additionally, *in vitro* infection with *Leishmania amazonensis* impairs human monocytes to differentiate into DCs, weakening the induction of a proper Th1 cell response (22).

Pondering that neutrophils and monocytes could interact at the site of *Leishmania* infection, we raised the question whether NETs could impact monocytes differentiation into DCs, affecting the response to parasites. Our results clearly show that NETs impair fully differentiation of monocytes into DCs and downregulate the expression of IL-4 receptor on monocytes due to elastase activity. NETs intimately interact with monocytes and IL-4/GM-CSF-treated monocytes exposed to NETs are less efficient in *Leishmania* killing. Moreover, IL-4/GM-CSF-treated monocytes cultured in the presence of NETs exhibited increased expression of molecules and cytokines associated with an anti-inflammatory macrophage phenotype. Our results suggest that NETs interfere with monocyte differentiation, reprogramming the generation of mo-DCs to an anti-inflammatory macrophage phenotype.

## Materials and Methods

### Neutrophil and Monocyte Purification

Human neutrophils and monocytes were isolated by density gradient centrifugation (Histopaque; Sigma-Aldrich) from buffy coats of healthy blood donors. PBMCs were collected and washed three times with PBS, resuspended in RPMI medium 1640 (Sigma-Aldrich) supplemented with Nutridoma-CS (1×; Roche Applied Science) and incubated for adherence (see below). In some experiments, monocytes were purified with the Monocyte Isolation Kit II (Miltenyi Biotec) according to the manufacturer’s instructions.

The layer containing neutrophils was subjected to hypotonic lysis of erythrocytes. Purified neutrophils were resuspended in RPMI medium 1640 and kept on ice until use. Human PBMCs from healthy subjects were obtained under written informed consent and all procedures were approved by the Institutional Review Board for Human Subjects (Research Ethics Committee) from Hospital Clementino Fraga Filho, Universidade Federal do Rio de Janeiro (protocol number 055-15) and from the NIH Clinical Center IRB-approved protocol from the NIH Clinical Center Department of Transfusion Medicine (protocol number 99-CC-0168).

### Monocyte-DC Differentiation Assay

PBMCs (5 × 10^6^; 1 mL) were incubated for 2 h at 37°C in 5% CO_2_ to allow monocytes to adhere in 24-well plates. Non-adherent cells were washed out and the adhered monocytes were used throughout the experiments. NETs-enriched supernatants treated or not with DNase, or filtered were added and cultures maintained at 37°C in 5% CO_2_. After 18 h, GM-CSF and IL-4 (50 ng/mL each; Peprotech) were added and the cultures maintained for 5 days at 37°C in 5% CO_2_. Cells were then harvested, stained for CD1a (PE; BD), CD14 (FITC; BD), CD68 (FITC; BD), CD32 (PE-Cy7; BD), CD163 (APC; BD), and CD80 (APC-Cy; BD), and analyzed on a MACSQuant flow cytometer (Miltenyi Biotec). Cells were acquired based on forward and side scatter and data analyzed with FlowJo Software 10.0.8. All experiments with monocytes were done in medium supplemented with Nutridoma-CS (1×; Roche Applied Science). In some experiments, monocytes were pretreated with cytochalasin D (CytD) (10 µg/mL; Sigma) or the diluent DMSO for 30 min before the addition of NETs.

### Parasite Culture

*Leishmania amazonensis* (WHOM/BR/75/Josefa) promastigotes were grown at 26°C in Schneider’s Insect Medium (Sigma-Aldrich), supplemented with 10% heat-inactivated fetal calf serum (FCS; Crypion, São Paulo, Brazil) and 40 µg/mL of gentamicin (Schering-Plough, Rio de Janeiro, Brazil). At days 5–6 of culture, stationary-phase promastigotes were obtained and used throughout the experiments. In parallel, *L. amazonensis* promastigotes were fixed with paraformaldehyde (4% in PBS) for 30 min at room temperature. Parasites were then extensively washed with PBS and resuspended in RPMI for further NET induction.

### Production of NETs-Enriched Supernatants

Neutrophils (8 × 10^6^) were incubated with live or paraformaldehyde-fixed promastigotes of *L. amazonensis* in a 1:0.1 neutrophil:parasite ratio or LPS (*Escherichia coli* O55:B5) 100 ng/mL at 35°C in 5% CO_2_. After 3 h incubation, supernatant was collected and aliquots were kept at −80°C until use. The quantification of NETs were performed with the Picogreen dsDNA kit (Invitrogen, Life Technologies), as already described (15). The NETs-enriched supernatants were treated with DNase (10 U/mL; Fermentas Life Science) or with the elastase inhibitor MeOSuc-AAPV-cmk (10 µg/mL, Calbiochem) for 30 min and then added to monocytes. In some cases, NETs were filtered through a 0.22 µm pore filter to remove the NET scaffold.

The quantification of the elastase in the supernatants was performed as described (18). Briefly, 50 µL of NETs-enriched supernatants were incubated with the fluorogenic substrate *N*-methoxysuccinyl-Ala-Ala-Pro-Val-7-amido-4-methylcoumarin (0.25 mM; Sigma-Aldrich). After 1 h at 37°C, the fluorescence was measured in a SpectraMax Paradigm reader (Molecular Devices) at 365–450 nm. A standard curve with recombinant elastase was used to determine the concentration of elastase in the NETs-enriched supernatants.

### Cell Viability Assay

Adhered monocytes were treated or not with NETs-enriched supernatants as described above for 18 h, and lactate dehydrogenase (LDH) in the culture supernatant was measured according to the manufacturer’s directions (Promega). Briefly, 50 µL of culture supernatant was incubated with 50 µL of substrate mix in a 96-well plate at room temperature protected from light. After 30 min, 50 µL of stop solution was added, and the plate was read at 490 nm on a SpectraMax Paradigm reader.

### Immunofluorescence

PBMCs (1 × 10^6^; 1 mL) were incubated for 2 h at 37°C in 5% CO_2_ to allow monocytes to adhere to coverslips. Non-adherent cells were washed out and adhered monocytes were incubated with NETs-enriched supernatants (2 µg of DNA) for 4 h and fixed with 4% paraformaldehyde. Slides were stained with DAPI (10 µg/mL; Sigma), anti-elastase (1:800 v/v; Calbiochem), or anti-histone/DNA complex (1:150 v/v; Abcam), followed by anti-rabbit-FITC (1:150 v/v; Vector Labs) or anti-rabbit-Texas Red (1:150 v/v; Invitrogen), respectively. Epifluorescence images were taken in a Zeiss Axioplan using 40× objectives.

### Phagocytosis and *Leishmania* Killing Assay

For the phagocytosis assay, promastigotes of *L. amazonensis* were labeled with the CellTrace™ CFSE Cell Proliferation Kit as recommended by the manufacturer (Invitrogen, Life Technologies). Briefly, promastigotes (1 × 10^6^ cells/mL) were incubated with 2.5 µM of CFSE in pre-warmed (37°C) PBS. After 10 min at 37°C, five volumes of ice-cold RPMI were added in order to quench the staining and cells were incubated for 5 min on ice. Parasites were then washed three times and resuspended in RPMI. Monocytes were treated as described in the Section “Monocyte-DC Differentiation Assay.” After 18 h of NET treatment, monocytes were incubated with CFSE-labeled *L. amazonensis* promastigotes at a 1 monocyte:3 parasites ratio. After 4 h of incubation at 37°C in 5% CO_2_, monocytes were harvested, washed and cells were analyzed on a FACSCalibur flow cytometer (Becton Dickinson). Monocytes were acquired based on forward and side scatter and data were analyzed with Summit Software 4.3. CFSE-positive monocytes were considered as infected cells.

For the parasite killing assay, adhered monocytes were incubated with NETs, treated or not with nuclease or elastase inhibitor for 18 h at 37°C in 5% CO_2_. Promastigotes were added to the culture in a 1 monocyte:3 parasites ratio and the coculture was maintained overnight at 35°C/5%CO_2_. Non-phagocytosed parasites were washed out. After 48 h of infection, monocytes were lysed with 0.01% sodium dodecyl sulfate (SDS) for 5 min at room temperature. Lysis was stopped by addition of Schneider medium supplemented with 10% FCS (Schneider complete medium), and parasites allowed to differentiate and grow for 48 h. Viable and motile promastigotes were counted in a Neubauer chamber.

### Cytokines Quantification

Monocytes were treated with NETs-enriched supernatants as described in the Section “Monocyte-DC Differentiation Assay.” After 5 days, cells were activated with LPS (100 ng/mL; Sigma) and cell culture supernatant was collected after 72 h and kept at −80°C until use. TNF-α, interleukin-12p40 (IL-12p40), transforming growth factor-β (TGF-β), and IL-10 concentrations were quantified by ELISA (Duo-Set Kits; R&D Systems) according to manufacturer’s instructions.

### Expression of IL-4 and GM-CSF Receptors

Adhered monocytes were incubated or not with NETs-enriched supernatants (2 µg of NET-DNA), treated or not with nuclease or the elastase inhibitor methoxysuccinyl-Ala-Ala-Pro-Val-chloromethyl ketone (MeOSuc-AAPV-cmk; Calbiochem) and cultures maintained at 37°C in 5% CO_2_. After 18 h, cells were harvested, incubated with FcR blocking reagent (Miltenyi), and stained with anti-IL-4 receptor α chain (PE; R&D) and anti-GM-CSF receptor (APC; R&D) antibodies. Cells were acquired based on forward and side scatter and analyzed using a FACSCalibur flow cytometer (BD) and data were analyzed with Summit Software 4.3.

For real-time PCR quantification of the expression of the IL-4 receptor, monocytes (1 × 10^6^ cells), purified with the Monocyte Isolation Kit II (Miltenyi Biotec), were treated or not with digested or non-digested NETs (dNETs)-enriched supernatants. After 4 h of treatment, RNA was extracted from the cell pellets, using the RNeasy mini Kit (Qiagen, Valencia, CA, USA) and treated with DNase I. cDNA synthesis was performed using the qScript cDNA Supermix (Quanta Biosciences, Gaithersburg, MD, USA). Genomic DNA contamination was measured by PCR of total RNA. Relative quantification of the IL-4 receptor expression was performed in a LightCycler 480 (Roche Applied Science, Indianapolis, IN, USA) using the Universal ProbeLibrary system (Roche). Primers (Left: CGTCTGCCTGTTGTGCTATG and Right: GGAATCTGATCCCACCATTC) and probe (Probe #9; cat 04685075001) were designed using ProbeFinder software Version 2.45 (Roche). Relative quantification analysis of the target gene versus 18 s was performed using the LightCycler 480 software.

### Western Blot Assay

IL-4 receptor cleavage was analyzed by Western blot. Recombinant soluble Human sIL-4 Receptor α (200 ng; Peprotech) was incubated with NETs (160 ng of NET-DNA), pretreated or not with 10 µg/mL of elastase inhibitor, or with recombinant elastase in different concentrations for 30 min at 37°C. Ten microliters were submitted to 10% SDS-PAGE. Western blot was carried using a Human IL-4 Rα biotinylated antibody (0.1 µg/mL; R&D) followed by avidin–alkaline phosphatase (1:150,000 v/v; Sigma-Aldrich) incubation. Blot was revealed with Western Blue stabilized substrate for alkaline phosphatase (Promega), according to manufacturer’s instructions.

### Data Analysis

Results are expressed as mean ± SEM and *P* < 0.05 was considered significant. For multiple comparisons, One-way ANOVA analysis followed by Fisher’s least significant difference was performed. Paired *t*-test analysis was performed for some experiments as indicated in the figure legend. Data analysis was performed by GraphPad Prism 5.03 software.

## Results

### NETs Impair Fully Maturation of Monocytes into DCs

**Figure 1:**
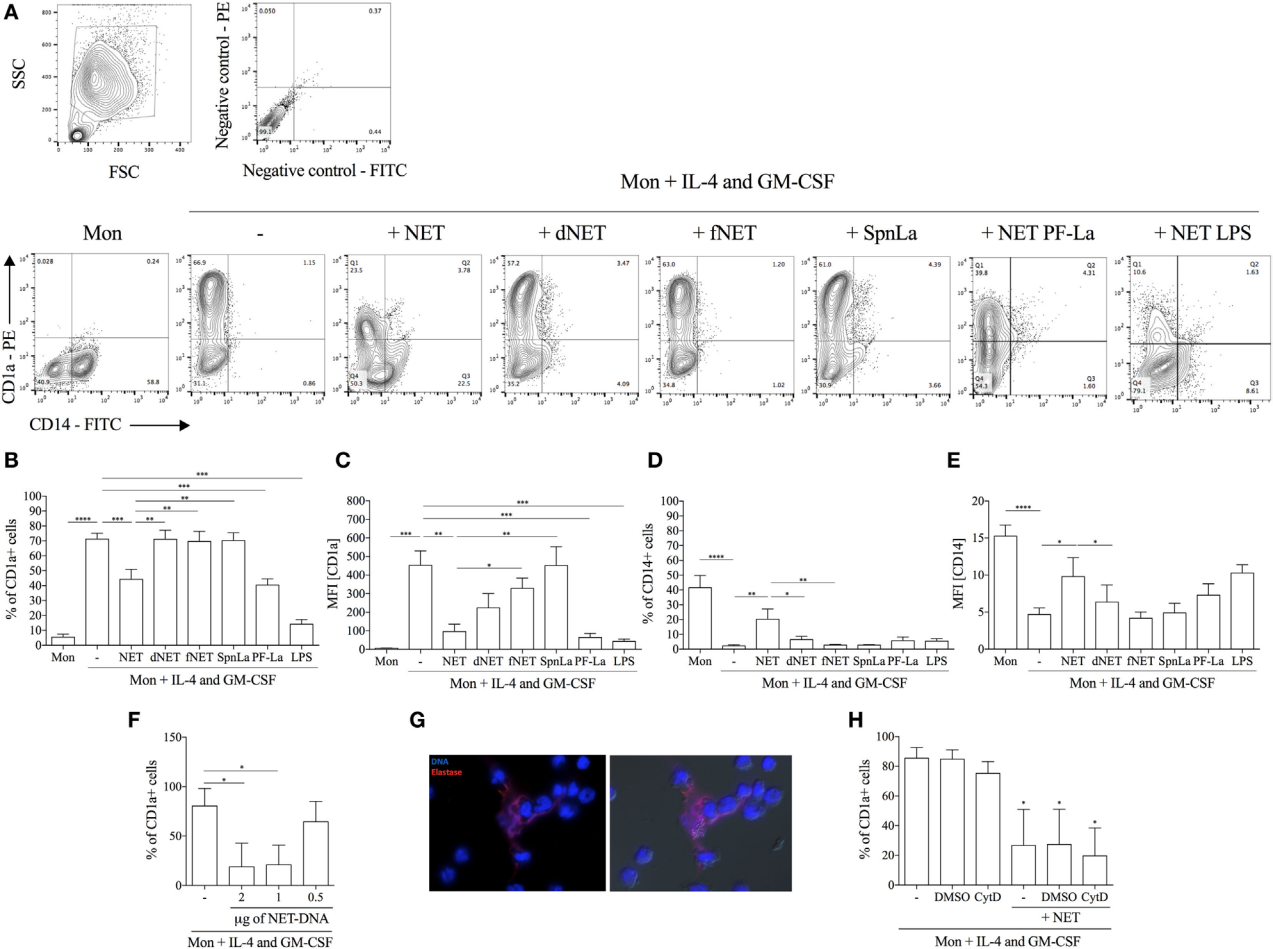
**Neutrophil extracellular traps (NETs) impair fully differentiation of monocytes into dendritic cells**. Adhered monocytes were incubated or not with NETs-enriched supernatants, which were treated or not with DNase, or filtered for 18 h. IL-4 and GM-CSF were added to the cultures (as indicated), which were maintained at 37°C/5%CO_2_. After 5 days, cells were harvested and stained for CD1a **(B,C)** and CD14 **(D,E)** and analyzed on a FACSCalibur flow cytometer. **(A)** Contour plots of one representative experiment showing the gate strategy. **(B–E)** The column Mon means that the adhered monocytes were not treated with NETs nor IL-4/GM-CSF. Digested NET (dNET) means supernatants enriched in NET pretreated with DNase (10 U/mL) 30 min before addition to monocytes. fNET means NET-free supernatants filtered through a 0.22 µm pore filter. PF-La and lipopolysaccharide (LPS) mean supernatants enriched in NET produced by paraformaldehyde killed parasites or LPS-activated neutrophils, respectively. Results of at least six independent experiments are shown as mean ± SEM. **(B)** ****, ***, **P < 0.01. **(C)** ***, **P < 0.01; *P < 0.05. **(D)** ****, **P < 0.01; *P < 0.05. **(E)** ****P < 0.01; *P < 0.05. **(F)** Adhered monocytes were incubated or not with different concentrations of NETs-enriched supernatants and the percentage of CD1a^+^ cells were analyzed as in panels **(A–E)**. Results of three independent experiments are shown as mean ± SEM. *P < 0.05 related to monocytes only incubated with IL-4/GM-CSF (first column). **(G)** Adhered monocytes were incubated with NETs-enriched supernatants for 4 h at 37°C/5%CO_2_. Cells were fixed and stained for DNA (blue) with 4′,6-diamidino-2-phenylindole and with anti-elastase followed by Texas red-labeled secondary antibody. Left image shows the overlay of DNA and elastase staining and right image shows the overlay of DNA and elastase staining with differential interference contrast image. **(H)** Monocytes were pretreated with cytochalasin D for 30 min before the addition of NETs and the percentage of CD1a^+^ cells were analyzed as in panels **(A–E)**. Results of four independent experiments are shown as mean ± SEM. *P < 0.01 related to monocytes only incubated with IL-4/GM-CSF (first column).

Neutrophil extracellular traps-enriched supernatants (here referred as NETs) were added to adhered monocyte culture 18 h before treatment with IL-4/GM-CSF, and differentiation of monocytes into DCs was analyzed by the surface membrane expression of CD1a and CD14 by flow cytometry. Human monocytes are CD14^+^CD1a^−^ (Figures 1A–E) and, during the IL-4/GM-CSF-driven *in vitro* differentiation process, their expression of CD14 decreased, whereas the expression of CD1a increased, as expected. Treatment with NETs before the addition of IL-4/GM-CSF, lead to a reduction in the percentage of CD1a^+^ cells by 42%, with a 79% reduction of the MFI (Figures 1B,C), relative to control (cells treated only with IL-4/GM-CSF). Expression of CD14 was partially maintained by NET treatment with 20% of CD14^+^ cells, a 7.7 times higher expression compared to control (2.6% of CD14^+^ cells) (Figure 1D). In addition, CD14 MFI increased 2.7 times in monocytes treated with NETs compared to control (Figure 1E). Moreover, the effect of NETs on monocyte differentiation is dose-dependent (Figure 1F), and treatment with DNase-dNETs or filtered-NETs had no effect on monocyte differentiation into DCs (Figures 1A–E). As additional controls, supernatants from promastigotes cultured in the same conditions used for NETs generation (SpnLa; Figure 1), and DNase added to monocyte cultures in the same conditions used to obtain dNETs (data not shown) had no effect on monocytes differentiation. To rule out the effect of any *Leishmania* molecule in the reprogramming of monocytes to DCs, we used NETs released by PF-fixed *Leishmania*-activated neutrophils (PF-La), and detected that NETs induced by fixed parasites were also able to inhibit DCs generation, likewise live *Leishmania*-induced NETs (Figures 1A–E). Importantly, NETs released by LPS-activated neutrophils also inhibited DCs generation from human monocytes (Figures 1A–E), suggesting that blockage of monocytes differentiation is not restricted to NETs generated by *Leishmania* activation of neutrophils.

To observe the cell interaction with NETs, monocytes were incubated during 4 h with these webs, washed, fixed, and NETs-associated DNA and elastase were characterized (Figure 1G). As portrayed, monocytes intimately interacted with NETs, which were visualized surrounding groups of monocytes. It is worthy to point out that NETs integrity was still maintained during the recovery of NETs-enriched supernatants. Furthermore, treatment with NETs-enriched supernatants was not toxic to monocytes, as assessed by extracellular LDH measurement (data not shown).

Because it has been reported that NETs interact and are endocytosed by human macrophages (23), we asked whether blockage of NETs uptake by monocytes would inhibit NETs effect on monocyte differentiation. Our results show that NETs were still able to impair mo-DCs generation even after endocytosis inhibition by CytD treatment (Figure 1H).

### NETs Downregulate IL-4 Receptor Expression

**Figure 2:**
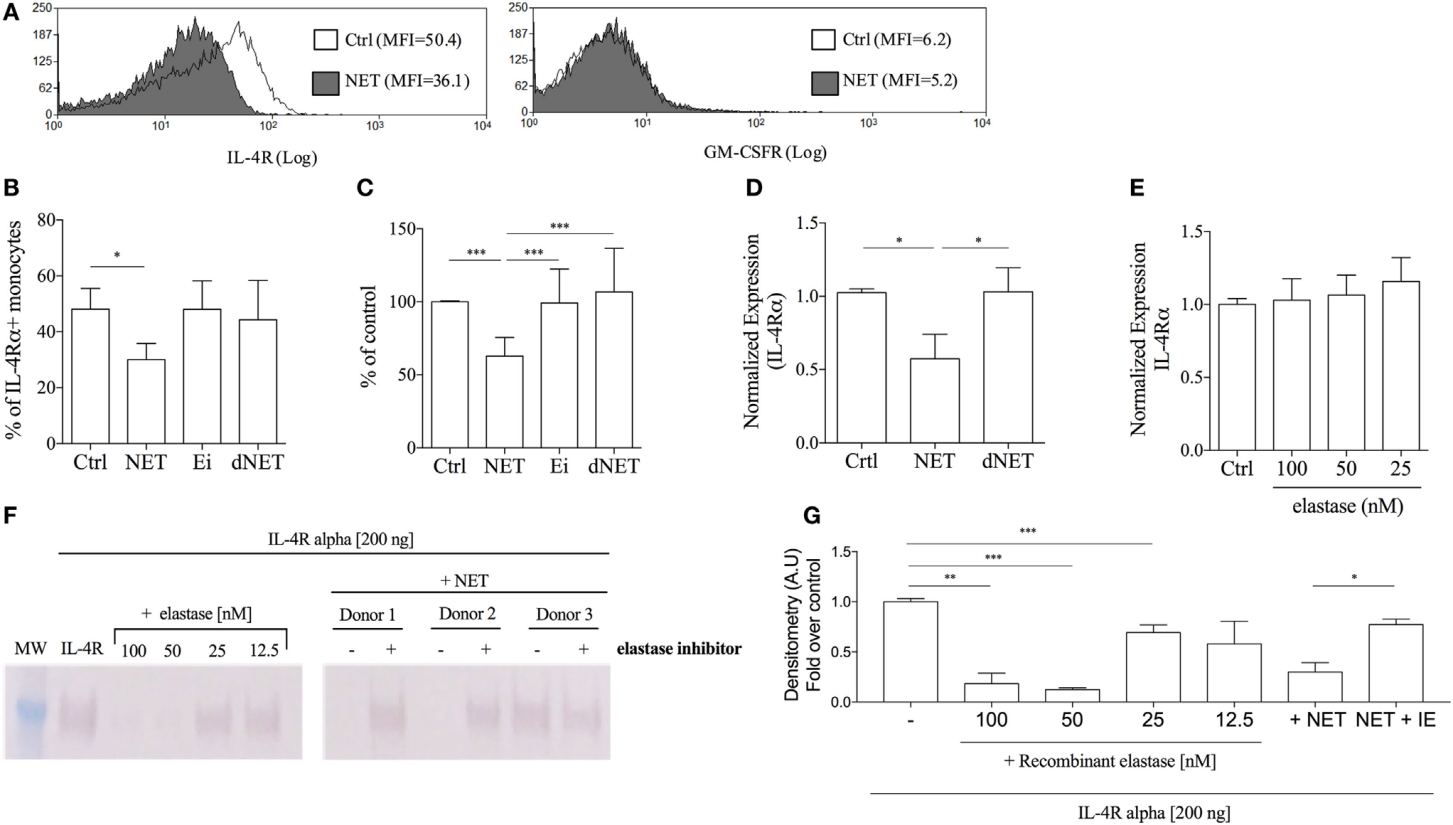
**Neutrophil extracellular traps (NETs) downregulates the expression of IL-4 receptor on monocytes**. **(A,B)** Adhered monocytes were incubated or not with NETs, which were treated or not with DNase, or elastase inhibitor for 18 h at 37°C/5%CO_2_. Cells were harvested and stained for the GM-CSF receptor and for the IL-4 receptor α chain after FcR blocking. **(A)** Histogram of one representative experiment. **(B,C)** Percentage of monocytes expressing the IL-4 receptor. Results of at least five independent experiments are shown as mean ± SEM. **(B)** Paired *t*-test analysis was performed and *P < 0.05 related to NETs untreated monocytes (control, ctrl). **(C)** ***P < 0.01. **(D,E)** Purified monocytes were treated or not with NETs-enriched supernatants, with digested NETs or NETs + elastase inhibitor or treated only recombinant elastase. After 4 h at 37°C/5%CO_2_, RNA was extracted and the cDNA synthesis was performed. Relative quantitative analysis of the target gene versus 18 s was performed using the LightCycler 480 software. Results of at least four independent experiments are shown as mean ± SEM. *P < 0.05 related to the control. **(F)** Recombinant soluble IL-4 receptor α (200 ng) was incubated with NETs (from three different donors), pretreated (+) or not (−) with 10 µg/mL of elastase inhibitor, or with recombinant elastase in different concentrations for 30 min at 37°C. IL-4 receptor cleavage was then analyzed by western blot. **(G)** Densitometric analysis of the IL-4Rα chain obtained from three independent experiments were analyzed by Image J. Results were normalized by the untreated recombinant IL-4R.

It was previously reported that neutrophil elastase cleaves G-CSF receptor (24). Since elastase is one of the NETs components, we asked whether NETs could also downregulate the expression of GM-CSF or IL-4 receptors, thus impairing the differentiation of monocytes into DCs. Interestingly, we found that the expression of IL-4 receptor (α chain) was reduced by 38% in NET-treated monocytes in comparison with control (Figures 2A–C). Pretreatment of NETs with elastase inhibitor rescued the expression of IL-4Rα (Figures 2B,C), suggesting a role for this enzyme in NETs-induced reduction of IL-4Rα expression. DNase-dNETs did not change IL-4Rα expression (Figures 2B,C), and we did not observe any differences in the expression of GM-CSF receptors in monocytes treated with NETs (Figure 2A). Next, by real-time PCR analysis of IL-4Rα mRNA expression, we tested whether NETs-induced downregulation of IL-4Rα expression could also occur at a transcriptional level. We found that monocytes treated with NETs express 50% less IL-4R mRNA than control (Figure 2D), suggesting that the IL-4R mRNA synthesis or transcriptional control is altered in NETs-exposed monocytes. IL-4R mRNA expression was not affected by DNase-dNETs (Figure 2D). Interestingly, monocytes treated with commercial recombinant elastase did not show any difference in IL-4R mRNA expression, relative to non-treated cells, showing that the preserved structure of NETs might be crucial for this activity (Figure 2E). To evaluate whether NETs could cleave the IL-4R, we incubated commercial IL-4Rα with different concentrations of elastase, NETs, or elastase inhibitor-treated NETs for 30 min. Our results show that elastase and NETs cleave IL-4R and that pretreatment of NETs with 10 µg/mL of elastase inhibitor prevented degradation of the receptor (Figures 2F,G).

### NETs-Treated Monocytes Differentiate into Macrophages

**Figure 3:**
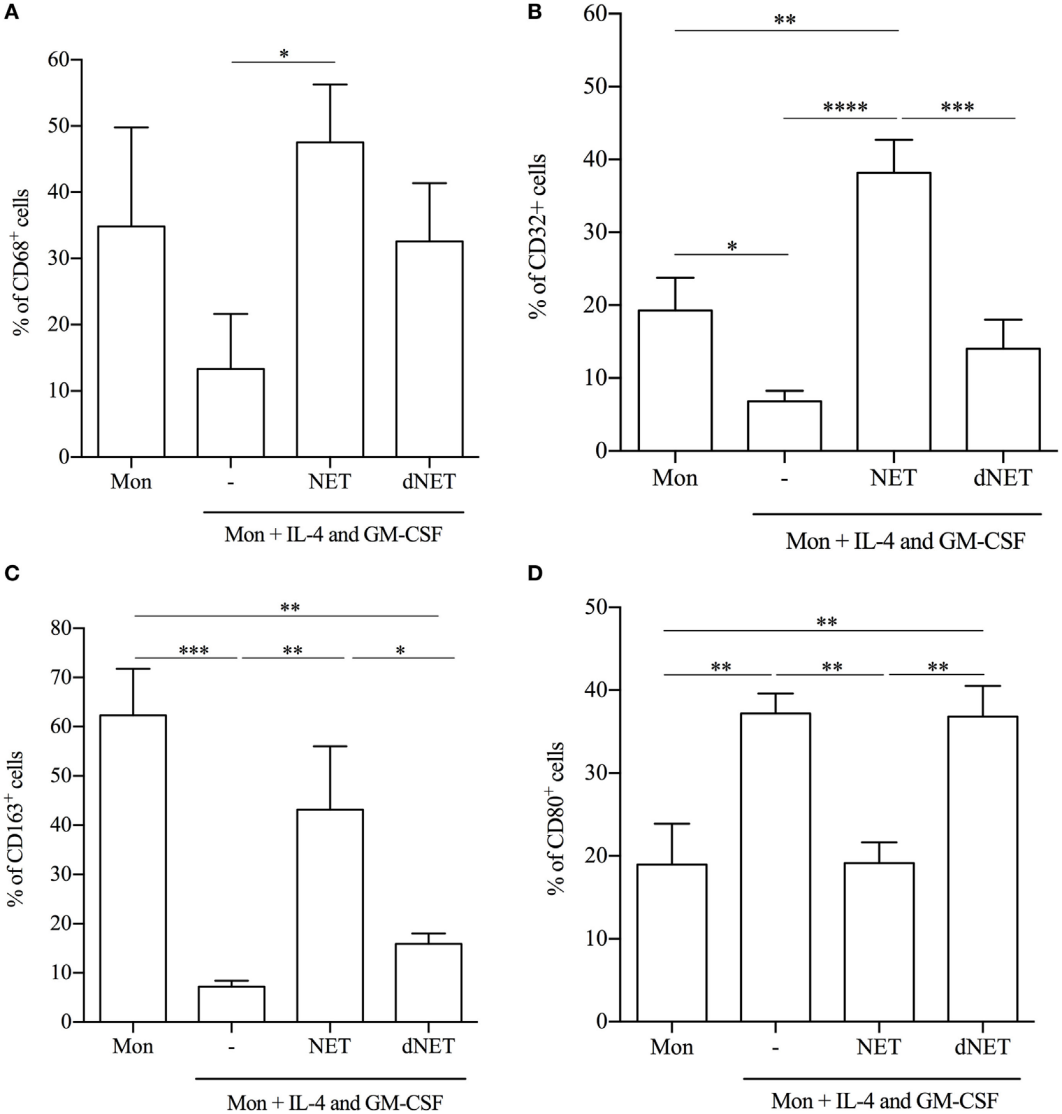
**Neutrophil extracellular traps (NETs)-treated monocytes differentiate into macrophages**. Adhered monocytes were incubated or not with NETs-enriched supernatants or digested NETs (dNETs) for 18 h. IL-4 and GM-CSF were added to the culture (where indicated) and the cells were maintained at 37°C/5%CO_2_. **(A–D)** After 5 days, cells were harvested and stained for **(A)** CD68, **(B)** CD32, **(C)** CD163, and **(D)** CD80. Results of at least five experiments are shown as % of cells expressing the cell marker and as mean ± SEM. **(A)** *P < 0.05. **(B)** ****, ***, **P < 0.001; *P < 0.05. **(C)** ***, **P < 0.01; *P < 0.05. **(D)** **P < 0.05.

Our results showing that monocytes treated with NETs before the addition of IL-4/GM-CSF were unable to fully differentiate into DCs led us to wonder whether these monocytes were being skewed into macrophages. Thus, we analyzed the expression of common surface and intracellular molecules of macrophages in the cells that emerged from the monocytes treated with NETs/IL-4/GM-CSF. We observed that NET-treated monocytes had an increased percentage of CD68^+^, CD32^+^, and CD163^+^ cells in comparison to monocytes cultured only with IL-4/GM-CSF (Figures 3A–C). However, NETs treatment diminished the percentage of CD80^+^ cells, suggesting that NETs can up- or downregulate monocyte membrane molecules. NETs disrupted by DNase treatment did not change the expression of CD32, CD80, and CD163 relative to IL-4/GM-CSF-treated cells (Figures 3B–D).

**Figure 4:**
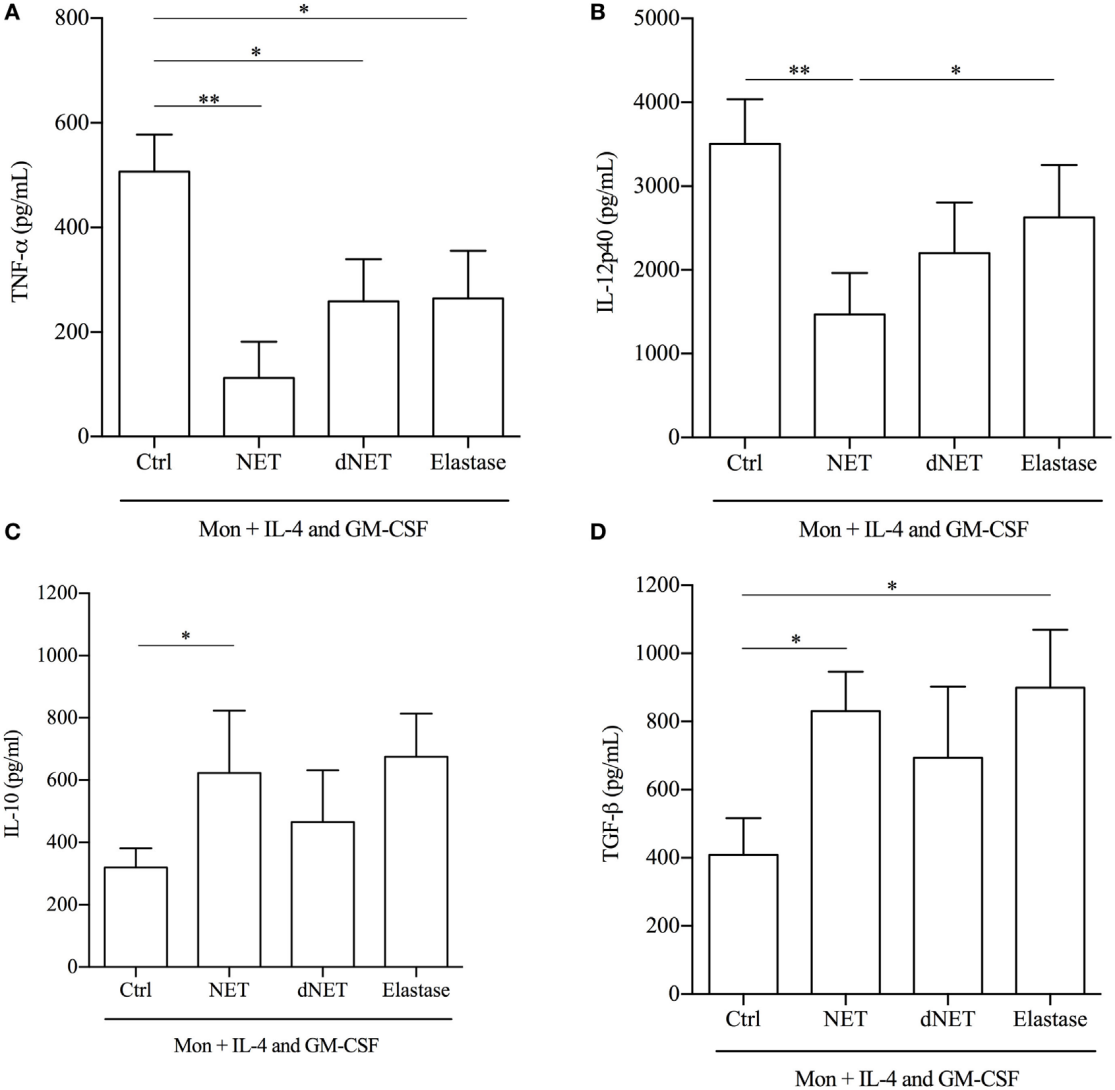
**Cytokines produced by neutrophil extracellular traps (NETs)-treated monocytes**. Monocytes were treated or not with NETs, digested NETs (dNETs), or recombinant elastase. After 18 h at 37°C/5%CO_2_, IL-4 and GM-CSF were added and cells were maintained at 37°C/5%CO_2_. At day 5, lipopolysaccharide (100 ng/mL) was added to the culture. After 72 h of activation, cytokine production was evaluated by ELISA. Results of at least eight experiments are shown as mean ± SEM. **(A)** **P < 0.01; *P < 0.05. **(B)** **P < 0.01; *P < 0.05. **(C)** Paired *t*-test analysis was performed and *P < 0.05. **(D)** *P < 0.05.

We also analyzed the secretion of cytokines by NET-treated cells, stimulated with LPS. Monocytes differentiated in the presence of NETs produced 78 and 70% less amounts of TNF-α and IL-12p40, respectively, than cells cultured with IL-4/GM-CSF only (Figures 4A,B). Although elastase and dNETs increased 2.3 times the amounts of TNF-α in relation to NETs/IL-4/GM-CSF-treated cells, they decreased around 49% TNF-α production in relation to control cells. Similarly, both elastase and dNETs treatments increased around two times IL-12p40 secretion in relation to NETs/IL-4/GM-CSF-treated cells, and decreased 25 and 37%, respectively, the IL-12p40 secretion in relation to control cells (Figures 4A,B). Inversely, NETs-treated monocytes produced significantly higher amounts (twice as much for each cytokine) of TGF-β and IL-10 (Figures 4C,D). Monocytes treatment with dNET or elastase did not change IL-10 production compared to control cells (Figure 4C). Elastase treatment increased 2.2 times TGF-β amounts in relation to control cells and no difference was observed comparing elastase and dNET in relation to NETs/IL-4/GM-CSF-treated cells (Figure 4D). Hence, we can conclude that NETs treatment reprograms the IL-4/GM-CSF-induced monocyte differentiation to monocytes with an anti-inflammatory profile.

### NETs-Treated Monocytes Are Less Efficient in Parasite Killing

**Figure 5:**
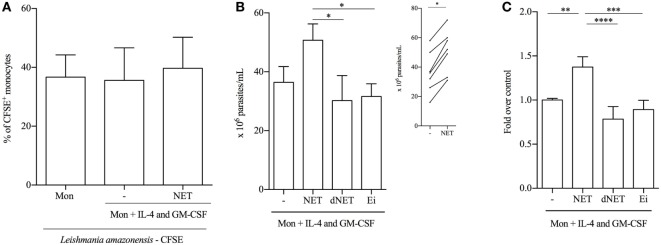
**Neutrophil extracellular traps (NETs)-treated monocytes are less efficient in parasite killing**. **(A)** Adhered monocytes were treated with NET for 18 h at 37°C/5%CO_2_. After 18 h of incubation, CFSE-labeled promastigotes of *Leishmania amazonensis* were added and cocultured at 35°C/5%CO_2_. Monocytes were then analyzed on a FACSCalibur flow cytometer (Becton Dickinson). CFSE^+^ cells were considered as monocytes that bound to or phagocytosed parasites. Results of six independent experiments are shown as mean ± SEM. **(B,C)** Adhered monocytes were treated with digested, elastase inhibitor-treated, or non-treated NETs or with *L. amazonensis* supernatant for 18 h at 37°C/5%CO_2_. Cells were then washed and promastigotes were added to the culture in a 1 monocyte:3 parasites ratio and cocultured at 35°C/5%CO_2_ overnight. After 48 h of infection, monocytes were lysed with 0.01% sodium dodecyl sulfate and Schneider medium supplemented with 10% FBS was added to the cultures to allow parasites grow. Viable and motile parasites were counted after 48 h in a Neubauer chamber. Results of five to seven independent experiments are shown as mean ± SEM. *P < 0.05. Insert shows the number of parasites in NET-treated and -untreated monocytes. In the insert graph, paired *t*-test analysis was performed and *P < 0.01. **(C)** Untreated monocytes (first column) were normalized and data are expressed as fold over the control. **(C)** ****, ***, **P < 0.01.

**Figure 6:**
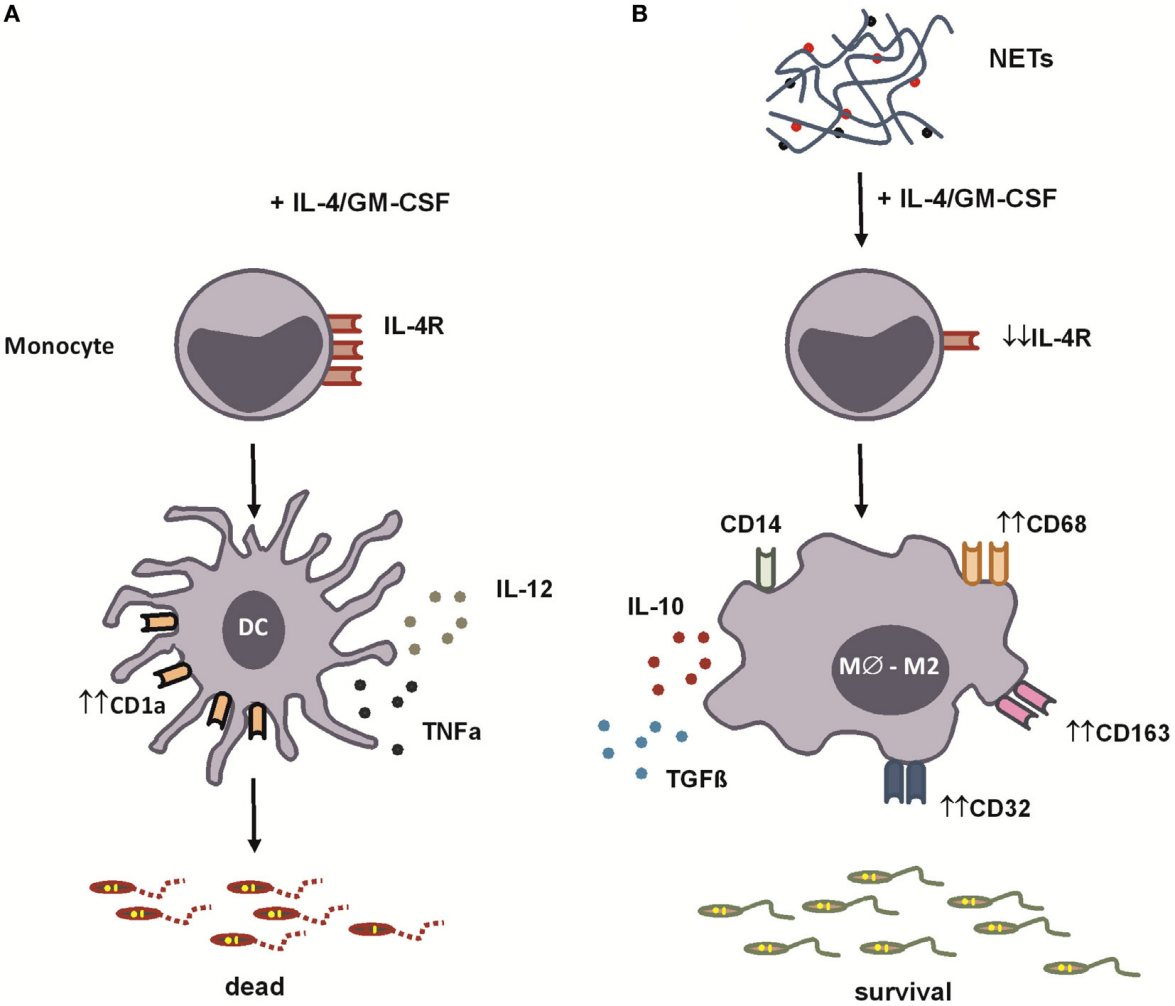
**Summary scheme for neutrophil extracellular traps’ (NETs’) blockage of dendritic cells (DCs) differentiation from monocytes**. **(A)** In the presence of IL-4 and GM-CSF, monocytes are stimulated to differentiate into DCs. These cells express CD1a molecules and, when stimulated by lipopolysaccharide (LPS), produce high amounts of pro-inflammatory cytokines [IL-12 and tumor necrosis factor-α (TNF-α)]. **(B)** We show in this study that NETs, produced by *Leishmania amazonensis*-activated human neutrophils, downregulate the expression of the IL-4 receptor α chain skewing monocytes to differentiate into anti-inflammatory macrophages. Macrophages differentiated in the presence of NETs express CD68, CD163, and CD32 and, when stimulated by LPS, produce high amounts of interleukin-10 (IL-10) and transforming growth factor-β (TGF-β), favoring *Leishmania* survival.

To study a functional aspect of the generated cells, we evaluated the antimicrobial capacity of both kinds of differentiated monocytes, by measuring their ability to phagocytose *Leishmania* promastigotes. NETs did not interfere with the ability of IL-4/GM-CSF-treated monocytes to bind parasites (Figure 5A). However, NETs-treated monocytes partially lost their capacity to kill parasites, compared to IL-4/GM-CSF-treated monocytes (Figures 5B,C). Importantly, this result was further confirmed in another set of experiments, in which NET treatment significantly decreased the ability to kill parasites of cells from seven different donors tested (Figure 5B, insert). The ability to kill parasites was preserved in dNETs- and elastase inhibitor-treated monocytes (Figures 5B,C). During the interaction with NETs-treated monocytes, parasite survival was equal to 70% higher, relative to survival rate observed in monocytes treated with dNETs (Figure 5C), meaning that disruption of NETs with DNase treatment and elastase inhibition rescued the ability of monocytes to kill *Leishmania* (Figures 5B,C). We show in Figure 6 a summary scheme of how NETs block DCs generation from monocytes.

## Discussion

Neutrophil extracellular traps have been extensively studied in the last decade. Since the first report, NETs have been suggested to participate in a great number of infectious and non-infectious diseases. It has been reported by our group and others that *Leishmania* parasites induce the release of NETs (15, 16, 25). We also demonstrated the presence of NETs structure in biopsies of patients with cutaneous leishmaniasis (15, 19). However, little is known about the impact of NETs on other immune cells, which prompted us to investigate whether NETs could affect human monocytes. Herein, we demonstrate that NETs downregulate the expression of the IL-4 receptor in monocytes, impairing the differentiation of these cells into immature DCs, and affecting killing of *Leishmania* parasites.

Impairing the generation of DCs derived from monocytes can prevent the generation of a protective immune response. DCs are the primary antigen-presenting cells and thereby connect the innate to the adaptive immune system. During infections, DCs are activated and enter into a process of maturation that involves changes in the ability to capture antigens, increased expression of costimulatory molecules, and migration to secondary lymphoid organs, where DCs activate T cells, controlling the quality of the Th1/Th2 immune response (26, 27). DCs play an important role in generating a protective Th1 response during *Leishmania* infection through the production of IL-12 (28), which is important for the infection control and the development of resistance. Th1 cells produce IFN-γ, which induces the expression of inducible nitric oxide synthase (iNOS) by phagocytic cells, leading to parasite killing (20). After migration through the endothelium, monocytes can differentiate into macrophages or DCs at the infection site (20). The mo-DCs are essential for the formation of a protective Th1 response during *Leishmania* infection. It has been shown that mo-DCs are formed at the site of *Leishmania* infection and constitute the main iNOS-producing cells, which is important for the elimination of parasites (20, 21). Nevertheless, during infection by *Leishmania mexicana*, the recruitment of monocytes to the site of infection is reduced and mo-DCs that are harvested from lesions produce low amounts of NO (29). Furthermore, it was demonstrated that the *in vitro* infection of human monocytes with promastigotes of *L. amazonensis* prevents the formation of mo-DCs, affecting the generation of a protective immune response (22). Our data demonstrate that NETs impair the process of monocytes differentiation into DCs and NETs digestion with DNase did not affect this differentiation process. The excessive NET formation at the site of *Leishmania* infection or a defect in the clearance of NET-structures could affect the generation of a protective Th1 response, which is important for the infection control. In fact, it has been demonstrated that during *L. mexicana* infection in C57BL6 mice, early neutrophil recruitment is associated with a decrease of monocyte and mo-DCs recruitment (25). The authors also showed the presence of NETs in *L. mexicana* mouse ear infection (25). Future studies need to be conducted to evaluate the impact of NETs in generating mo-DCs in the *in vivo* model of *Leishmania* infection and its implication in the development of a Th1 immune response.

A likely explanation for the impairment in monocytes differentiation process is the modulation of the expression of receptors for GM-CSF and IL-4. It has been demonstrated that treatment of human neutrophils with recombinant neutrophil elastase decreases the expression of G-CSF receptor in a time-dependent fashion, as this enzyme can cleave G-CSFR (24). Digestion of the receptor was observed by detection of receptor’s fragments in culture of elastase-treated neutrophils (24). Since elastase is one of the major components of NETs, we investigated its effect in the expression of IL-4 and GM-CSF receptors. Interestingly, a decrease in the IL-4Rα expression was detected on NETs-treated monocytes, together with a decrease in the IL-4R mRNA, indicating that NETs induce not only digestion of the expressed receptor, as well as, exert a transcriptional regulation of this receptor. We have not detected any changes in the expression of GM-CSFR. Digestion of NETs fully abolished the elastase effect on the expression of the IL-4R on monocytes, indicating that NET structure must be intact to be effective. Moreover, this data indicates that degranulated neutrophil elastase would not have the same effect as NETs-associated elastase, since the structural integrity of NETs is required to regulate IL-4R expression. We have not established here the mechanism by which NETs downregulate IL-4R expression. However, it was demonstrated that type I IFN and IFN-γ diminish IL-4R expression on mononuclear cells and B cells purified from human peripheral blood (30). It was also demonstrated that both types of IFN post-transcriptionally downregulate IL-4R expression by affecting the mRNA stability (30). Besides, IFN-γ reduces the expression of both γc and α IL-4R chains on human monocytes cell surface (31). NETs were previously shown to be the major inducer of type I IFN production by plasmacytoid DCs during autoimmune disease (5), a possible explanation for NETs’ downregulation of the IL-4R would be through the production of IFN by NETs-treated monocytes.

The lower expression of the α chain of the IL-4R may have implications in the course of the disease. The protective immune response during *Leishmania* infection requires the activation of Th1 cells and production IFN-γ, which activate the macrophages’ microbicidal mechanisms. By contrast, the Th2 response with high production of IL-4 and IL-13 is associated with susceptibility to infection. BALB/c mice are susceptible to infection by *L. major* due to the development of a Th2 response to the parasite. BALB/c mice deficient in IL-4 or with the expression of IL-4Rα deleted specifically in CD4^+^ T cells become resistant to *Leishmania* infection (32, 33). In addition, treatment of *L. major*-infected mice with soluble IL-4R results in a decrease in parasite load in spleen and lymph nodes and promoted resistance to reinfection by *L. major* (34).

Despite the deleterious effect of IL-4 during *Leishmania* infection, contrasting data have been reported in the literature regarding the role of IL-4 during infection. Unexpectedly it has been demonstrated that administration of recombinant IL-4 8 h after infection with *L. major* promotes IL-12 production by DCs *in vivo* (35). However, when IL-4 was administered at later time points, C57BL/6 resistant mice become susceptible to *L. major* infection (35). These data demonstrate that IL-4 has different roles during the course of leishmaniasis. This interleukin seems to have a protective effect in the early post-infection events. Furthermore, deletion of the α chain of the IL-4 receptor on DCs turn BALB/c hypersusceptible to infection with *L. major* (36), indicating a protective role of IL-4 during *Leishmania* infection. All these studies show that the role of IL-4 in *Leishmania* remains contradictory. In our study, we have not evaluated the production of IL-4 by monocytes treated with NETs. However, these cells expressed lower amounts of IL-4Rα on its surface. The absence of IL-4 signaling pathway in monocytes specifically has not yet been evaluated during *Leishmania* infection. Our data point to a lower parasite killing capacity of monocytes cultured in the presence of NETs. Further studies needs to be conducted to correlate the expression of the IL-4R on monocytes treated with NETs with the monocyte microbicidal capacity.

Monocytes that were treated with NETs differentiated more in macrophages than in DCs, with expression of CD68, a macrophage marker, and classic macrophage morphology (a network of elongated cells with strongly adherence to plastic; data not shown). The analysis of surface markers of differentiated cells in the presence of NETs revealed that NETs-treated monocytes differentiated into macrophages with a profile of CD32^+^CD68^+^CD163^+^CD1a^−^ cells. Moreover, these cells have reduced expression of CD80 and increased expression of CD163 when treated with NETs, thereby characterizing an M2 macrophage, as established in the literature (37, 38). Studies in the literature suggest that NETs activate a pro-inflammatory response in other immune cells. These structures are capable of activating the production of type I IFN in plasmacytoid DCs and activate the production of pro-inflammatory cytokines (such as IL-1β, TNF-α, and IL-6) in macrophages (5–7, 10). However, different from what it was shown in the literature, in our experiments NETs-treated monocytes secreted more anti-inflammatory (TGF-β and IL-10) than pro-inflammatory (TNF-α and IL-6) cytokines. Accordingly, our data also show that NETs-treated monocytes presented a lower parasite killing capacity and generate M2 macrophages, suggesting that NETs might be activating an anti-inflammatory immune response in these cells.

Despite the ability of neutrophils to release traps in response to parasite presence, a portion of the population can escape NET-mediated killing. Parasites that have escaped NETs will infect other cells, such as inflammatory recruited monocytes, macrophages, and DCs. Upon reaching the site of infection, monocytes interact with NETs and parasites. We hypothesize that NETs decrease the differentiation of monocytes into DCs and at the same time disables the microbicidal mechanisms of these monocytes, which differentiate into M2 macrophages. The data presented here provide new evidence about the role of NETs in *Leishmania* infection. The reduced expression of IL-4R caused by NETs may result in modulation of the immune response to the parasite and thereby alter the course of the disease. Furthermore, the effect in reducing differentiation of monocytes and decreasing the expression of IL-4 identified in this work can be applied to any other disease or inflammatory event where the interaction between neutrophils and monocytes occurs and is relevant. Hence, the relevance of the data presented here is not restricted to infection by the parasite *Leishmania*.

## Ethics Statement

Human PBMCs from healthy subjects were obtained under written informed consent and all procedures were approved by the Institutional Review Board for Human Subjects (Research Ethics Committee) from Hospital Clementino Fraga Filho, Universidade Federal do Rio de Janeiro (protocol number 055-15) and from the NIH Clinical Center IRB-approved protocol from the NIH Clinical Center Department of Transfusion Medicine (protocol number 99-CC-0168).

## Author Contributions

Conceived and designed the experiments and wrote the paper: AG-C, FO, JE-L, and ES. Performed the experiments: AG-C and NR. Analyzed the data: AG-C, NR, FO, JE-L, and ES.

## Conflict of Interest Statement

The authors declare that the research was conducted in the absence of any commercial or financial relationships that could be construed as a potential conflict of interest.

## References

[1] Brinkmann V Reichard U Goosmann C Fauler B Uhlemann Y Weiss DS Neutrophil extracellular traps kill bacteria. Science (2004) 303:1532–5. 10.1126/science.1092385 15001782

[2] Urban CF Ermert D Schmid M Abu-Abed U Goosmann C Nacken W Neutrophil extracellular traps contain calprotectin, a cytosolic protein complex involved in host defense against *Candida albicans*. PLoS Pathog (2009) 5(10):e1000639. 10.1371/journal.ppat.1000639 19876394 PMC2763347

[3] Sangaletti S Tripodo C Chiodoni C Guarnotta C Cappetti B Casalini P Neutrophil extracellular traps mediate transfer of cytoplasmic neutrophil antigens to myeloid dendritic cells toward ANCA induction and associated autoimmunity. Blood (2012) 120(15):3007–18. 10.1182/blood-2012-03-416156 22932797

[4] Leffler J Martin M Gullstrand B Tydén H Lood C Truedsson L Neutrophil extracellular traps that are not degraded in systemic lupus erythematosus activate complement exacerbating the disease. J Immunol (2012) 188(7):3522–31. 10.4049/jimmunol.1102404 22345666

[5] Lande R Gregorio J Facchinetti V Chatterjee B Wang YH Homey B Plasmacytoid dendritic cells sense self-DNA coupled with antimicrobial peptide. Nature (2007) 449(7162):564–9. 10.1038/nature06116 17873860

[6] Lande R Ganguly D Facchinetti V Frasca L Conrad C Gregorio J Neutrophils activate plasmacytoid dendritic cells by releasing self-DNA-peptide complexes in systemic lupus erythematosus. Sci Transl Med (2011) 3(73):73ra19. 10.1126/scitranslmed.3001180 21389263 PMC3399524

[7] Garcia-Romo GS Caielli S Vega B Connolly J Allantaz F Xu Z Netting neutrophils are major inducers of type I IFN production in pediatric systemic lupus erythematosus. Sci Transl Med (2011) 3(73):73ra20. 10.1126/scitranslmed.3001201 21389264 PMC3143837

[8] Kessenbrock K Krumbholz M Schönermarck U Back W Gross WL Werb Z Netting neutrophils in autoimmune small-vessel vasculitis. Nat Med (2009) 15(6):623–5. 10.1038/nm.1959 19448636 PMC2760083

[9] Hakkim A Fürnrohr BG Amann K Laube B Abed UA Brinkmann V Impairment of neutrophil extracellular trap degradation is associated with lupus nephritis. Proc Natl Acad Sci U S A (2010) 107(21):9813–8. 10.1073/pnas.0909927107 20439745 PMC2906830

[10] Braian C Hogea V Stendahl O. *Mycobacterium tuberculosis*-induced neutrophil extracellular traps activate human macrophages. J Innate Immun (2013) 5(6):591–602. 10.1159/000348676 23635526 PMC6741595

[11] Warnatsch A Ioannou M Wang Q Papayannopoulos V Neutrophil extracellular traps license macrophages for cytokine production in atherosclerosis. Science (2015) 349(6245):316–20. 10.1126/science.aaa8064 26185250 PMC4854322

[12] Barrientos L Bignon A Gueguen C de Chaisemartin L Gorges R Sandré C Neutrophil extracellular traps downregulate lipopolysaccharide-induced activation of monocyte-derived dendritic cells. J Immunol (2014) 193(11):5689–98. 10.4049/jimmunol.1400586 25339673

[13] Peters NC Egen JG Secundino N Debrabant A Kimblin N Kamhawi S In vivo imaging reveals an essential role for neutrophils in leishmaniasis transmitted by sand flies. Science (2008) 321(5891):970–4. 10.1126/science.1159194 18703742 PMC2606057

[14] Ribeiro-Gomes FL Peters NC Debrabant A SackS DL. Efficient capture of infected neutrophils by dendritic cells in the skin inhibits the early anti-*Leishmania* response. PLoS Pathog (2012) 8(2):e1002536. 10.1371/journal.ppat.1002536 22359507 PMC3280984

[15] Guimarães-Costa AB Nascimento MT Froment GS Soares RP Morgado FN Conceição-Silva F *Leishmania amazonensis* promastigotes induce and are killed by neutrophil extracellular traps. Proc Natl Acad Sci U S A (2008) 106(16):6748–53. 10.1073/pnas.0900226106 PMC2672475 19346483

[16] Gabriel C McMaster WR Girard D Descoteaux A. *Leishmania donovani* promastigotes evade the antimicrobial activity of neutrophil extracellular traps. J Immunol (2010) 185(7):4319–27. 10.4049/jimmunol.1000893 20826753

[17] Guimarães-Costa AB DeSouza-Vieira TS Paletta-Silva R Freitas-Mesquita AL Meyer-Fernandes JR Saraiva EM. 3′-nucleotidase/nuclease activity allows *Leishmania* parasites to escape killing by neutrophil extracellular traps. Infect Immun (2014) 82(4):1732–40. 10.1128/IAI.01232-13 24516114 PMC3993383

[18] Chagas AC Oliveira F Debrabant A Valenzuela JG Ribeiro JM Calvo E. Lundep, a sand fly salivary endonuclease increases *Leishmania* parasite survival in neutrophils and inhibits XIIa contact activation in human plasma. PLoS Pathog (2014) 10(2):e1003923. 10.1371/journal.ppat.1003923 24516388 PMC3916414

[19] Morgado FN Nascimento MT Saraiva EM de Oliveira-Ribeiro C Madeira Mde F da Costa-Santos M Are neutrophil extracellular traps playing a role in the parasite control in active American tegumentary leishmaniasis lesions? PLoS One (2015) 10(7):e0133063. 10.1371/journal.pone.0133063 26192752 PMC4508047

[20] Leon B Lopes-bravo M Ardavin C. Monocyte-derived dendritic cells formed at the infection site control the induction of protective T helper 1 responses against *Leishmania*. Immunity (2007) 26(4):519–31. 10.1016/j.immuni.2007.01.017 17412618

[21] De Trez C Magez S Akira S Ryffel B Carlier Y Muraille E. iNOS-producing inflammatory dendritic cells constitute the major infected cell type during the chronic *Leishmania major* infection phase of C57BL/6 resistant mice. PLoS Pathog (2009) 5(6):e1000494. 10.1371/journal.ppat.1000494 19557162 PMC2695779

[22] Favali C Tavares N Clarêncio J Barral A Barral-Netto M Brodskyn C. *Leishmania amazonensis* infection impairs differentiation and function of human dendritic cells. J Leukoc Biol (2007) 82(6):1401–6. 10.1189/jlb.0307187 17890507

[23] Farrera C Fadeel B. Macrophage clearance of neutrophil extracellular traps is a silent process. J Immunol (2013) 191(5):2647–56. 10.4049/jimmunol.1300436 23904163

[24] Piper MG Massullo PR Loveland M Druhan LJ Kindwall-Keller TL Ai J Neutrophil elastase downmodulates native G-CSFR expression and granulocyte-macrophage colony formation. J Inflamm (Lond) (2010) 7(1):5. 10.1186/1476-9255-7-5 20205821 PMC2824667

[25] Hurrell BP Schuster S Grün E Coutaz M Williams RA Held W Rapid sequestration of *Leishmania mexicana* by neutrophils contributes to the development of chronic lesion. PLoS Pathog (2015) 11(5):e1004929. 10.1371/journal.ppat.1004929 26020515 PMC4447405

[26] Rossi M Young JW. Human dendritic cells: potent antigen-presenting cells at the crossroads of innate and adaptive immunity. J Immunol (2005) 175(3):1373–81. 10.4049/jimmunol.175.3.1373 16034072

[27] Moser M Murphy KM. Dendritic cell regulation of Th1-Th2 development. Nat Immunol (2000) 1(3):199–205. 10.1038/79734 10973276

[28] Gorak PM Engwerda CR Kaye PM Dendritic cells, but not macrophages, produce IL-12 immediately following *Leishmania donovani* infection. Eur J Immunol (1998) 28:687–95. 10.1002/(SICI)1521-4141(199802)28:02<687::AID-IMMU687>3.0.CO;2-N 9521079

[29] Petritus PM Manzoni-DE-Almeida D Gimblet C Gonzalez Lombana C Scott P. *Leishmania mexicana* induces limited recruitment and activation of monocytes and monocyte-derived dendritic cells early during infection. PLoS Negl Trop Dis (2012) 6(10):e1858. 10.1371/journal.pntd.0001858 23094119 PMC3475671

[30] So EY Park HH Lee CE IFN-γ and IFN-α posttranscriptionally down-regulate the IL-4-induced IL-4 receptor gene expression. J Immunol (2000) 165(10):5472–9. 10.4049/jimmunol.165.10.5472 11067899

[31] Bonder CS Davies KV Hosszu EK Finlay-Jones JJ Hart PH IFN-γ downregulates interleukin-4 functional activity on monocytes by multiple mechanisms. J Interferon Cytokine Res (2002) 22(3):287–93. 10.1089/107999002753675703 12034035

[32] Kopf M Brombacher F Köhler G Kienzle G Widmann KH Lefrang K IL-4-deficient BALB/c mice resist infection with *Leishmania major*. J Exp Med (1996) 184(3):1127–36. 10.1084/jem.184.3.1127 9064329 PMC2192785

[33] Radwanska M Cutler AJ Hoving JC Magez S Holscher C Bohms A Deletion of IL-4Rα on CD4 T cells renders BALB/c mice resistant to *Leishmania major* infection. PLoS Pathog (2007) 3(5):e68 10.1371/journal.ppat.0030068 17500591 PMC1867380

[34] Gessner A Schröppel K Will A Enssle KH Lauffer L Röllinghoff M. Recombinant soluble interleukin-4 (IL-4) receptor acts as an antagonist of IL-4 in murine cutaneous leishmaniasis. Infect Immun (1994) 62(10):4112–7. 7927664 10.1128/iai.62.10.4112-4117.1994 PMC303084

[35] Biedermann T Zimmermann S Himmelrich H Gumy A Egeter O Sakrauski AK IL-4 instructs TH1 responses and resistance to *Leishmania major* in susceptible BALB/c mice. Nat Immunol (2001) 2:1054–60. 10.1038/ni725 11600887

[36] Hurdayal R Nieuwenhuizen NE Revaz-Breton M Smith L Hoving JC Parihar SP Deletion of IL-4 receptor alpha on dendritic cells renders BALB/c mice hypersusceptible to *Leishmania major* infection. PLoS Pathog (2013) 9(10):e1003699. 10.1371/journal.ppat.1003699 24204259 PMC3812013

[37] Martinez FO Sica A Mantovani A Locati M Macrophage activation and polarization. Front Biosci (2008) 13:453–61. 10.2741/2692 17981560

[38] Jaguin M Houlbert N Fardel O Lecureur V. Polarization profiles of human M-CSF-generated macrophages and comparison of M1-markers in classically activated macrophages from GM-CSF and M-CSF origin. Cell Immunol (2013) 281(1):51–61. 10.1016/j.cellimm.2013.01.010 23454681

